# Unlocking the energy capabilities of micron-sized LiFePO_4_

**DOI:** 10.1038/ncomms8898

**Published:** 2015-08-03

**Authors:** Limin Guo, Yelong Zhang, Jiawei Wang, Lipo Ma, Shunchao Ma, Yantao Zhang, Erkang Wang, Yujing Bi, Deyu Wang, William C. McKee, Ye Xu, Jitao Chen, Qinghua Zhang, Cewen Nan, Lin Gu, Peter G. Bruce, Zhangquan Peng

**Affiliations:** 1 State Key Laboratory of Electroanalytical Chemistry, Changchun Institute of Applied Chemistry, Chinese Academy of Sciences, Changchun, Jilin 130022, China.; 2 University of Chinese Academy of Sciences, Beijing 100049, China.; 3 Ningbo Institute of Materials Technology and Engineering, Chinese Academy of science, Ningbo, Zhejiang 315201, China.; 4 Department of Chemical Engineering, Louisiana State University, Baton Rouge, Louisiana 70803, USA.; 5 Beijing National Laboratory for Molecular Sciences, College of Chemistry and Molecular Engineering, Peking University, Beijing 100871, China.; 6 School of Materials Science and Engineering, State Key Lab of New Ceramics and Fine Processing, Tsinghua University, Beijing 100084, P.R. China.; 7 Beijing National Laboratory for Condensed Matter Physics, Institute of Physics, Chinese Academy of Sciences, Beijing 100190 China.; 8 Departments of Materials and Chemistry, University of Oxford, Parks Road, Oxford OX1 3PH UK.

## Abstract

Utilization of LiFePO_4_ as a cathode material for Li-ion batteries often requires size nanonization coupled with calcination-based carbon coating to improve its electrochemical performance, which, however, is usually at the expense of tap density and may be environmentally problematic. Here we report the utilization of micron-sized LiFePO_4_, which has a higher tap density than its nano-sized siblings, by forming a conducting polymer coating on its surface with a greener diazonium chemistry. Specifically, micron-sized LiFePO_4_ particles have been uniformly coated with a thin polyphenylene film via the spontaneous reaction between LiFePO_4_ and an aromatic diazonium salt of benzenediazonium tetrafluoroborate. The coated micron-sized LiFePO_4_, compared with its pristine counterpart, has shown improved electrical conductivity, high rate capability and excellent cyclability when used as a ‘carbon additive free' cathode material for rechargeable Li-ion batteries. The bonding mechanism of polyphenylene to LiFePO_4_/FePO_4_ has been understood with density functional theory calculations.

## Abstract (web summary)

Nanonization of battery electrode particles is a usual way to enhance their conductivity, but the decreased tap density is detrimental to battery performance. Here, the authors coat micron-sized lithium iron phosphate with a conducting polymer layer and demonstrate some excellent electrochemical properties.

## Graphical abstract

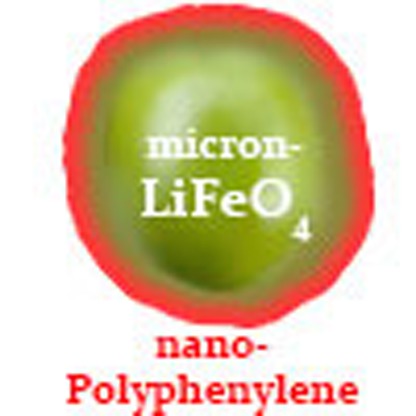

Li-ion battery, since its first commercialization in 1991, has drastically transformed and popularized portable electronic devices, and will continue to play a major role in the electrification of road transportation in the future^1^. However, for the realization of the latter, better energy storage materials are needed^1^,^2^,^3^,^4^,^5^,^6^,^7^,^8^,^9^,^10^,^11^,^12^,^13^. LiFePO_4_, an environmentally benign and relatively safe cathode material for rechargeable Li-ion batteries, has attracted a great deal of interest during the last few decades^2^,^3^,^4^. Considerable efforts have been devoted to overcoming the intrinsically low electrical conductivity of LiFePO_4_, a drawback that hinders its direct use in Li-ion cells^5^,^6^. Several strategies, such as doping with foreign metal ions, have been explored^7^,^8^,^9^. However, the most common approach remains coating with carbon^8^. Carbon coatings are usually formed during LiFePO_4_ synthesis, in which an organic precursor (the carbon source) and the inorganic raw materials are mixed together. The subsequent calcination of the mixture in an inert or reducing atmosphere produces conducting carbon and LiFePO_4_, simultaneously^10^,^11^,^12^. Similarly, carbon coatings can also be introduced after LiFePO_4_ synthesis, in which an organic precursor and preformed LiFePO_4_ are mixed and then calcined^13^,^14^. The calcination-based strategies are often energy intensive and can be environmentally unfriendly because of the emission of harmful volatile organic compounds from the thermal decomposition of organic precursors^15^. Moreover, carbon coatings on LiFePO_4_ produced by heat treatment tend to be irregular, which does not provide a good connectivity for the particles and hence the expected performance for battery applications^16^. To mitigate the negative environmental effects of calcination, conducting polymers have been employed to increase the electronic conductivity and thus improve the performance of LiFePO_4_ (refs 17, 18, 19, 20, 21, 22). Several methods have been used to produce polymer/LiFePO_4_ composites, including electrochemical^19^ and chemical^20^ polymerization in the presence of LiFePO_4_ particles; rapid mixing of conducting polymer colloidal and LiFePO_4_ suspensions^21^; and more recent spontaneous polymerization driven by the oxidation power of partially delithiated LiFePO_4_ (ref. 22).

It shall be noted here that the above mentioned carbon and conducting polymer-coating procedures work well mainly on nano-sized LiFePO_4_, ranging typically from 200 to 20 nm (ref. 3). The reason for using nano-sized LiFePO_4_ lies in that reducing particle size can shorten the solid-state diffusion distance within LiFePO_4_, which is beneficial to the high-power (or high rate) applications^23^. However, one obvious drawback associated with nano-sized LiFePO_4_ is the decreased tap density (and the resultant lower volumetric energy density), which becomes critical when fitting LiFePO_4_-based batteries into the trunks of pure electric vehicles^3^. Although the literatures on LiFePO_4_ are predominantly based on nano-sized materials, there are indeed some efforts of exploring submicron- and micron-sized LiFePO_4_. For instance, Dominiko *et al*.^24^ showed that a 0.5-μm carbon-free LiFePO_4_ has a specific capacity of 72 mAh g^−1^ at 1 C; McNeil *et al*.^25^ reported that a carbon-coated LiFePO_4_ (0.5–1.0 μm) exhibited a specific capacity of 129 mAh g^−1^ at 1 C; Wang *et al*. reported that 0.5 μm LiFePO_4_ could exhibit an initial capacity of 151 mAh g^−1^ at 1 C and 58 mAh g^−1^ at 10 C, but with limited cyclability^26^,^27^; larger (>5 μm) LiFePO_4_ particles have been identified to have very poor performance even when coated with conducting carbon^25^,^26^. So far, no facile procedure has been reported to make high-performance micron-sized LiFePO_4_, possibly due to the limited robustness of the coatings that cannot keep the integrity of the LiFePO_4_ particles during discharge and charging, particularly at high rates^27^.

Here we report a room-temperature method that can spontaneously coat micron-sized (∼1.01 μm) LiFePO_4_ uniformly with a thin conducting polymer of polyphenylene, as depicted in Fig. 1. The coated LiFePO_4_, compared with its pristine counterpart, has demonstrated enhanced electrical conductivity, high rate capability and excellent cyclability when used as a ‘carbon additive free' cathode material for rechargeable Li-ion batteries. In addition, the bonding of polyphenylene to LiFePO_4_, Li_1−*x*_FePO_4_ and FePO_4_ has been explored by performing density functional theory (DFT) calculations for the adsorption of the phenyl radical on model surfaces of these compounds. It is concluded that phenyl preferentially forms a strong chemical bond to surface O sites under typical experimental and battery operational conditions, which could be disrupted in the unlikely event that the surface of LiFePO_4_ becomes completely lithiated.

## Results

### Reaction of LiFePO_4_ and C_6_H_5_N_2_^+^BF_4_^−^

It is known that when an aromatic diazonium salt (ArN_2_^+^ X^−^) is subjected to electrochemical or chemical reduction or thermal decomposition, an aryl radical (Ar^·^) will form, which is reactive and is an effective agent for surface functionalization of many kinds of substrates^28^. The obtained organic layers strongly adhere to the substrates because a covalent bond is thought to form between the substrate surface and the aryl radicals^29^,^30^,^31^,^32^. Although most of the organic layers reported in the literatures are insulating^28^, a few of them are indeed conducting when certain diazonium precursors are used^31^,^33^,^34^. One such diazonium salt is benzenediazonium tetrafluoroborate (C_6_H_5_N_2_^+^BF_4_^−^), which can be easily and economically prepared in a single-step synthesis^31^ (Supplementary Figs 1 and 2 for the nuclear magnetic resonance (NMR) and electrospray ionization mass spectrometry (ESI-MS) characterization) and is employed here as the radical-generating agent to functionalize the micron-sized pristine LiFePO_4_. Powder X-ray diffraction (PXRD), scanning electron microscopy (SEM)/transmission electron microscopy (TEM) and Fourier transform infrared (FTIR) characterizations (Supplementary Figs 3–5) showed that the micron-sized LiFePO_4_ is of pure phase and contains no adventitious impurities such as Li_2_CO_3_ and LiOH.

**Figure 2:**
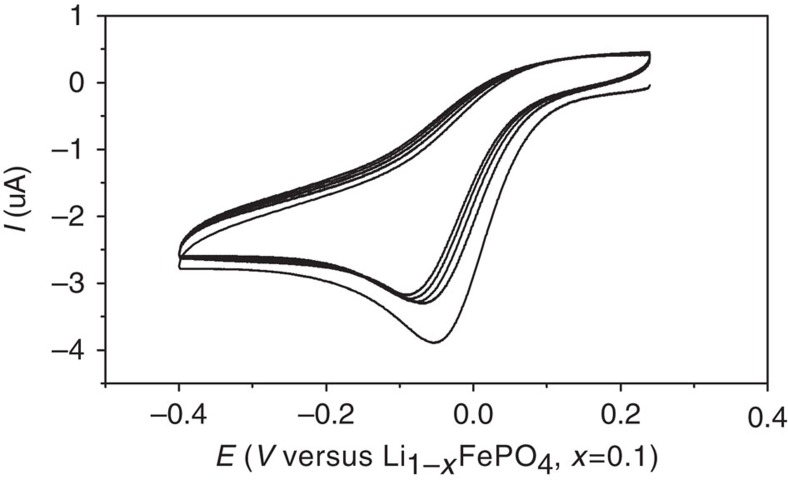
Measurement of the reduction potential of C_6_H_5_N_2_^+^BF_4_^−^. Electroreduction of 1 mM C_6_H_5_N_2_^+^BF_4_^−^ at a 2-mm diameter Au electrode in a three-compartment cell thermostated at 21 °C. Supporting electrolyte is 0.1 M TBAClO_4_-acetonitrile and scan rate is 0.1 V s^−1^.

To demonstrate the feasibility of the spontaneous reaction of LiFePO_4_ and C_6_H_5_N_2_^+^BF_4_^−^, an electrochemical measurement was performed. In a three-compartment cell, C_6_H_5_N_2_^+^ BF_4_^−^ is electrochemically reduced at an Au electrode, where partially delithiated LiFePO_4_ (that is, Li_1−*x*_FePO_4_, *x*=0.1) is used as the reference electrode because of its highly stable potential of 3.43 V versus Li^+^/Li^35^. Figure 2 shows the cyclic voltammograms for the first 1–5 cycles. One distinct feature of the *I*–*E* curves is that the successive peak currents corresponding to the electroreduction of C_6_H_5_N_2_^+^ to C_6_H_5_^·^ radical does not decrease drastically, which is different from the insulating film-forming diazonium salts, such as 4-nitrobenzenediazonium tetrafluoroborate^36^, a benchmark compound for electrografting of diazonium salt. This observation is consistent with the conducting nature of the grafted polyphenyl layers on the electrode^31^. Although the reduction peak potential is located at −0.05 V, the onset potential of the electroreduction of C_6_H_5_N_2_^+^ BF_4_^−^ is around 0.1 V, which is positive to the Li_1−*x*_FePO_4_ reference. It should be noted that the open circuit potential of the pristine LiFePO_4_ electrode against Li^+^/Li is in the range of 2.5–3.0 V, thus providing an even greater driving force for the reduction of C_6_H_5_N_2_^+^BF_4_^−^. Recently, Madec *et al*.^37^ have used nitrobenzenediazonium salts to functionalize pristine LiFePO_4_ and limited reaction extent has been observed, which could be due to the insulating nature of the polymers formed from the nitrobenzenediazonium precursors.

**Figure 3:**
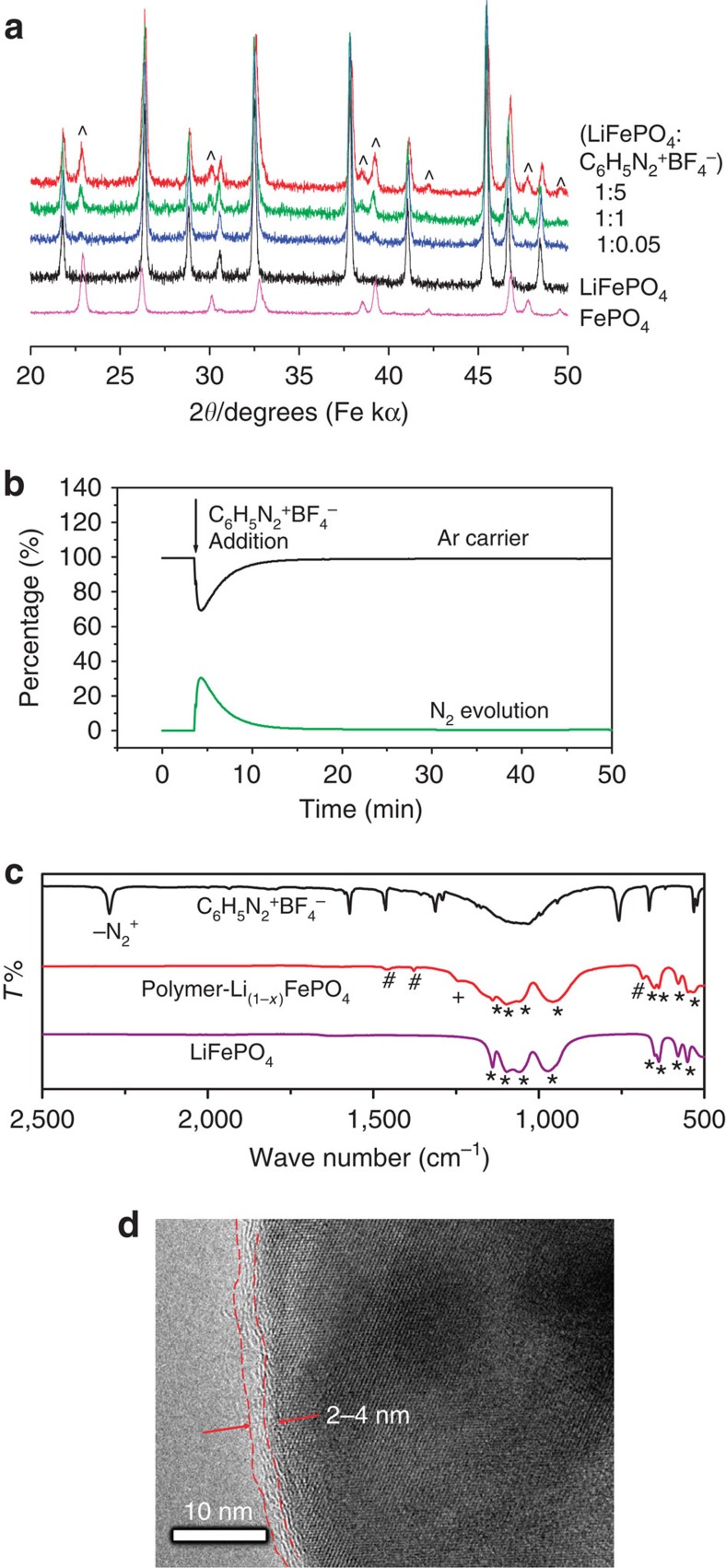
Characterizations of the reaction of LiFePO_4_ and C_6_H_5_N_2_^+^BF_4_^−^. (**a**) PXRD patterns of the reaction products of LiFePO_4_ and C_6_H_5_N_2_^+^BF_4_^−^ at different molar ratios (1:5, 1:1 and 1:0.05), together with pure phase LiFePO_4_ and FePO_4_. The symbol ^ highlights the evolution of FePO_4_ phase. (**b**) Quantitative online mass spectrometric analysis of N_2_ gas evolution of the reaction of 0.162 g LiFePO_4_ and 10 mg C_6_H_5_N_2_^+^BF_4_^−^. (**c**) FTIR of the reaction products of LiFePO_4_ and C_6_H_5_N_2_^+^BF_4_^−^ with molar ratio of 1:0.05, together with the spectra of LiFePO_4_ and C_6_H_5_N_2_^+^BF_4_^−^. The symbols *, + and # denote bands associated with LiFePO_4_, FePO_4_ and polyphenylene, respectively. (**d**) TEM of polyphenylene-coated LiFePO_4_.

Following the realization that LiFePO_4_ can reduce C_6_H_5_N_2_^+^ BF_4_^−^ to the C_6_H_5_^·^ radical, three reactions were conducted with molar ratios of LiFePO_4_:C_6_H_5_N_2_^+^BF_4_^−^ of 1:5, 1:1 and 1:0.05, respectively. The obtained products were subjected to structural and compositional analysis with the aim of understanding the extent and kinetics of the reaction. PXRD analysis (Fig. 3a) of the products after 12 h of reaction shows that the reaction, as expected, produces a new phase, that is, FePO_4_, and that the amount of the FePO_4_ increases with that of the added C_6_H_5_N_2_^+^ BF_4_^−^. However, the excess C_6_H_5_N_2_^+^ BF_4_^−^ cannot completely transform LiFePO_4_ to FePO_4_, which means the reaction could be a self-limiting process. At molar ratios of LiFePO_4_:C_6_H_5_N_2_^+^BF_4_^−^ of 1:5 and 1:1, around 48 and 44% of the pristine LiFePO_4_ were oxidized to FePO_4_, respectively, as determined by a Rietveld refinement procedure^38^ (Supplementary Fig. 6 and Table 1 for details). These two products contain a high ratio of FePO_4_, which is not beneficial to the direct application as cathode material of Li-ion battery because of the severe Li^+^ ion loss. However, the reaction of LiFePO_4_ and C_6_H_5_N_2_^+^ BF_4_^−^ with molar ratio of 1:0.05 shows a very small amount of FePO_4_ is formed (4.3% as measured indirectly by element analysis and online mass spectrometry, instead of Rietveld refinement due to the very weak PXRD signal of FePO_4_ phase, see Supplementary Fig. 6 and Table 1 for the fitting results) and thus much less Li is extracted, as seen in Fig. 3a (blue curve). It is this reaction with less C_6_H_5_N_2_^+^ BF_4_^−^ that we will focus on.

First we conducted a quantitative online mass spectrometric investigation (the technical details may be found in refs 39, 40) of the reaction of LiFePO_4_ and C_6_H_5_N_2_^+^ BF_4_^−^. In a typical experiment, 0.162 g of LiFePO_4_ was dispersed in acetonitrile in a reaction vial that was incorporated into the purging system of an online mass spectrometer. Before mixing with C_6_H_5_N_2_^+^ BF_4_^−^, only the signal of the Ar carrier gas was observed. On addition of C_6_H_5_N_2_^+^BF_4_^−^ solution (containing 10 mg of the diazonium salt as indicated by the arrow in Fig. 3b), the signal of N_2_ increased abruptly and the reaction finished within 20 min. The evolved N_2_ gas was quantified, according to a procedure published previously^39^,^40^, to be 1.03 ml (0.046 mmol), while the expected N_2_ volume was 1.15 ml (0.051 mmol). This discrepancy may be due to minor side reactions that do not release N_2_ gas^41^. To verify the formation of the conducting polyphenyl polymers on LiFePO_4_, the solid product was analysed with FTIR. Figure 3c shows the FTIR spectra of the LiFePO_4_ after reaction, and also the pristine LiFePO_4_ (associated bands are marked with *) and C_6_H_5_N_2_^+^BF_4_^−^ for comparison purposes. The band in the 2,300–2,130 cm^−1^ region corresponding to the stretching of the N≡N^+^ bond of the C_6_H_5_N_2_^+^ BF_4_^−^ is not present in the solid product after reaction, which confirms the loss of N_2_ during the reaction, consistent with the online mass spectrometric results. In addition, a new band at 1,240 cm^−1^ (marked with + in Fig. 3c, red curve) found in the spectrum of the solid product after reaction indicates the formation of FePO_4_ (refs 42, 43). The bands at 1,456 and 1,375 cm^−1^, corresponding to the stretching of C=C bonds in aromatic rings, together with a stronger band at 686 cm^−1^ (marked with # in Fig. 3c, red curve) associated with different types of aromatic substitutions^44^, indicate the formation of polyphenyl polymers. The different types of substitutions reflect the non-regiospecific attack of aryl radicals at those sites where the molecules are already attached to the surface, which has been found previously in the multilayers from diazonium reactions^31^,^36^.

Figure 3d shows the TEM micrograph of the polyphenylene-coated LiFePO_4_, in which a coating with a thickness of 2–4 nm has been observed for isolated particles. The formation of the polymer coating has also been proved by a high-angle annular scanning transmission electron microscopy equipped with a energy-dispersive X-ray detector for the elemental mapping of a single LiFePO_4_ particle after reaction (Supplementary Fig. 7). Although the PXRD data (Fig. 3a, blue curve) of the LiFePO_4_ after reaction with diazonium salt show a very weak signal of FePO_4_, the co-existence of two phases of LiFePO_4_ and FePO_4_ within a single particle (Supplementary Fig. 8) has been observed by annular bright-field scanning TEM^45^. We also noticed that the extended exposure of the polymer under electron beam causes degradation. Similar phenomena have been observed previously for polyaniline-coated noble metal particles^46^. The weight percentage of polyphenylene in the composite was determined by elemental analysis to be 2.0% (wt%), which is consistent with the value measured by online mass spectrometry 2.1% (wt%). The latter is transformed from a 4.3% (mol%) of LiFePO_4_ that is oxidized to FePO_4_, by taking into account that the reaction occurs according to LiFePO_4_+PhN_2_^+^→FePO_4_+Ph·+Li^+^+N_2_ and that the generated Ph· forms polymers on LiFePO_4_ surfaces. The formation of conducting polymer was further evidenced by the conductivity measurement of pressed powders, in which the polyphenylene-coated samples showed an electronic conductivity of 0.03 S cm^−1^, while pristine LiFePO_4_ is less than 10^−6^ S cm^−1^ (refs 7, 8). Higher conductivity of polyphenylene has previously been reported by Shacklette *et al*.^47^, who demonstrated that the conductivity of polyphenylene can be increased to 50 S cm^−1^ by doping with K^+^, and even to 500 S cm^−1^ by doping with AsF_5_^−^.

### Electrochemical performance

**Figure 4:**
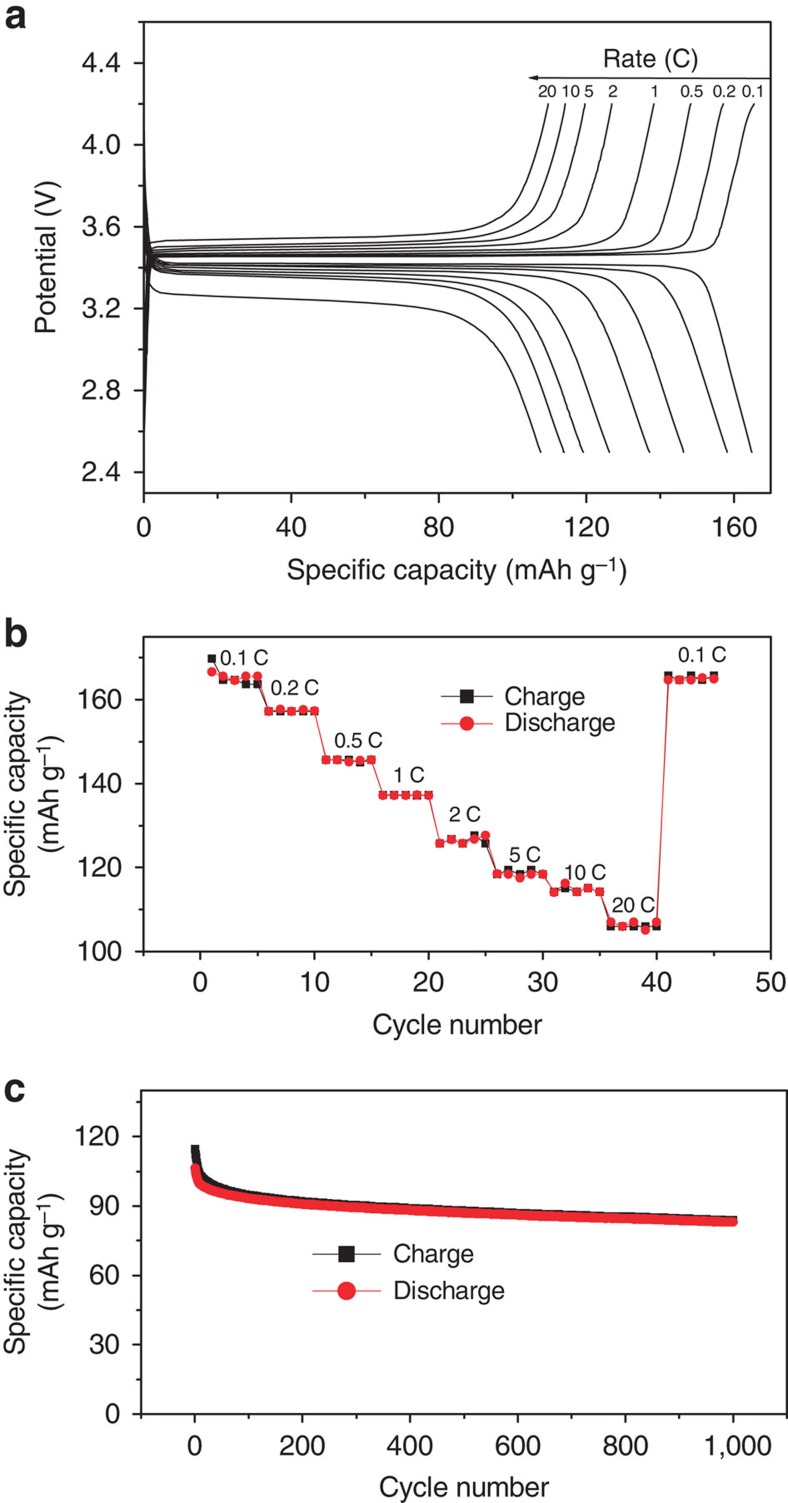
Electrochemical performance of polyphenylene-LiFePO_4_ composites. (**a**) Charge/discharge curves of polyphenylene-LiFePO_4_/PVDF (9:1 wt/wt) at various rates from 0.1 to 20 C; (**b**) charge/discharge capacity versus cycle number; (**c**) high rate performance at 20 C.

The obtained polymer/LiFePO_4_ composite had a tap density of 2.02 g cm^−3^ that was higher than the values reported for nano-sized LiFePO_4_ (typically in the range of 1.0–1.5 g cm^−3^), and were mixed with polyvinylidene difluoride (PVDF) binder (9:1 wt/wt) and casted on an Al foil current collector to make a cathode (mass loading in the range of 2–3 mg cm^−2^) for Li-ion cells. It is worth noting that the cathode does not contain any conducting carbon such as Super P, an additive that is extensively used in the practical cathode fabrication to ensure electronic conductivity throughout the electrode. When tested at a lower rate of 0.1 C, a capacity of 165 mAh g^−1^ (the capacity is normalized to the expected mass of the active material of the cathode after complete lithiation) was achieved, as seen in Fig. 4a,b. This value is close to the theoretical capacity of LiFePO_4_ (170 mAh g^−1^), while very limited capacity is obtained for LiFePO_4_ treated in the absence of C_6_H_5_N_2_^+^BF_4_^−^ (Supplementary Fig. 9). When the carbon additive of Super P was used, improved electrochemical performance, relative to that of pristine LiFePO_4_, has been obtained (Supplementary Fig. 10), which was, however, still inferior to the performance of the polyphenylene coating, particularly at higher rates. Electrochemical impedance spectroscopy has been used to probe the interfacial reaction kinetics, and smaller interfacial reaction resistance has been identified for polyphenylene-coated LiFePO_4_ (Supplementary Fig. 11), which could account for the improved performance at higher rates. The Li^+^ diffusion within the micron-sized LiFePO_4_ has also been measured with the electrochemical impedance spectroscopy, and diffusion coefficients in the range of 10^−16^–10^−14^ cm^2^ s^−1^ comparable to that of nano-LiFePO_4_ have been obtained^48^ (Supplementary Fig. 11 and Table 2). The performance of polyphenylene-coated LiFePO_4_ is significant since the prepared cathode contains no conducting carbon additive, and it shall be emphasized here that conducting carbon additive is not electrochemically active and therefore diminishes the practical energy density of the electrode^13^. The ability to replace carbon with a conducting polyphenylene coating is for these reasons highly advantageous. Cycling at a higher rate of 20 C for 1,000 cycles confirms the stability of the coating in the lithium-ion battery environment (Fig. 4c). Performances of the polyphenylene-coated LiFePO_4_ at different temperatures have also been tested, and the results at 0 °C demonstrated comparable performance at rates <8 C and somewhat degraded performance at rates >12 C (Supplementary Fig. 12). The high rate capability of the micron-sized LiFePO_4_ can be attributed to the formation of a metastable phase that can effectively decrease the energy battier of the nucleation and growth of a new phase, even the intrinsic Li^+^ bulk diffusion coefficient of LiFePO_4_ is small^49^,^50^.

### The interaction between LiFePO_4_/FePO_4_ and phenyl radicals

To address the issue of how polyphenylene is bonded to LiFePO_4_, we have performed DFT calculations using the phenyl radical as the probe molecule. As noted above, the initial reaction between LiFePO_4_ and C_6_H_5_N_2_^+^BF_4_^−^ can partially delithiates the former leading to the formation of lithium vacancies predominantly present on the surface region^51^. Furthermore, as previous studies have shown, typical discharge processes do not entirely convert FePO_4_ into LiFePO_4_ (ref. 52). Thus, we conclude that the surface region of LiFePO_4_ under typical experimental and operational conditions is best described either as FePO_4_ or as LiFePO_4_ with Li vacancies. The stoichiometric, fully lithiated LiFePO_4_ is included below for comparison purposes. The (010) termination is chosen because it is the dominant facet based on Wulff constructions for both LiFePO_4_ and FePO_4_ (ref. 53).

**Figure 5:**
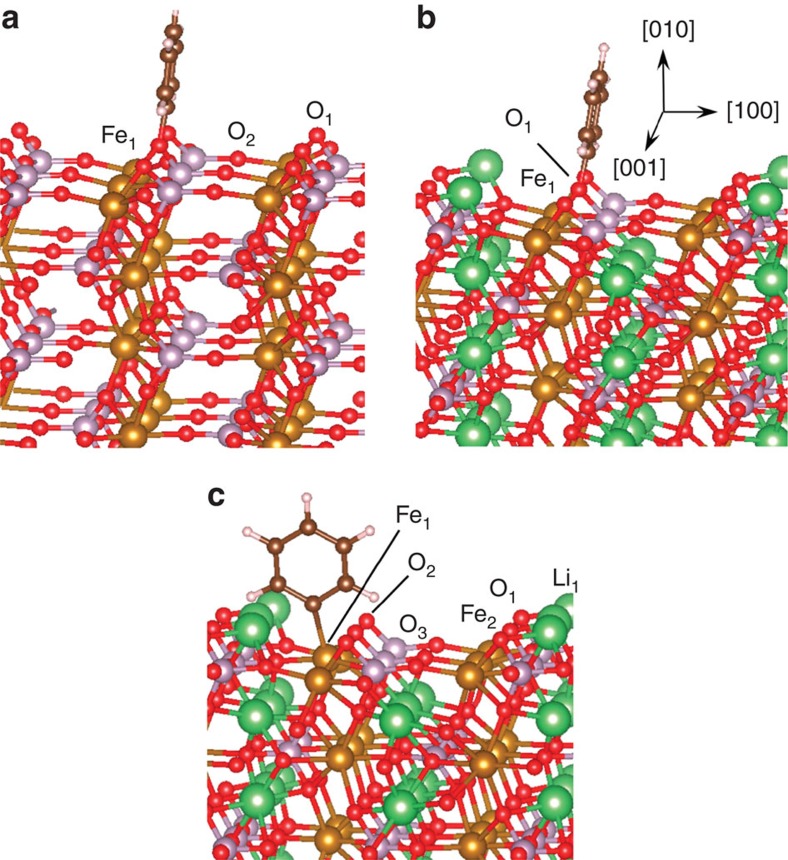
DFT-calculated configurations of phenyl radical on Li_1−*x*_FePO_4_(010). Minimum energy adsorption configurations for a phenyl radical on (**a**) FePO_4_(010), (**b**) LiFePO_4_(010) with a surface Li vacancy and (**c**) stoichiometric LiFePO_4_(010). The white, green, black, red, purple and gold spheres represent H, Li, C, O, P and Fe atoms, respectively. The orientation of all of the surface models is the same and is indicated in **b**.

On FePO_4_(010), phenyl is most stable on the O_1_ site (Fig. 5a) with a C–O bond length (*d*_C–O_) of 1.420 Å, which is consistent with covalent C–O single bonds. The adsorption energy (Δ*E*_ads_) is −2.80 eV, indicating the bond to be chemical in nature. We have not located any previous report of the phenyl adsorption energy on metal oxides for comparison. DFT-calculated Δ*E*_ads_ of phenyl on transition metal surfaces range widely from −1.04 (on Au(111)) to −2.81 (on Ti(0001)) eV^54^. Van der Waals forces may further stabilize phenyl adsorption^55^, but are not expected to change the site preference of phenyl on these surfaces. Phenyl adsorption on LiFePO_4_(010) with a Li vacancy is weaker than on FePO_4_(010), although phenyl still preferentially binds to an O site (Fig. 5b) with a considerably exothermic Δ*E*_ads_ of −1.55 eV and *d*_C–O_=1.414 Å. For comparison, phenyl adsorption on stoichiometric LiFePO_4_(010) has a Δ*E*_ads_ of only −0.18 eV (most stable O site is O_2_, Fig. 5c) with *d*_C–O_=1.410 Å.

In contrast, the adsorption of phenyl on Fe sites varies much less across the three model surfaces. Phenyl on the best Fe site (Fe_1_, Fig. 5a) has *d*_C–Fe_=2.239 Å and Δ*E*_ads_=−0.59 eV on FePO_4_(010); 2.113 Å and −0.84 eV on LiFePO_4_(010) with a Li vacancy (Fe_1_, Fig. 5b), and 2.136 Å and −0.94 eV, respectively, on stoichiometric LiFePO_4_(010) (Fe_1_, Fig. 5c).

Our DFT results suggest that the phenyl radical (and thus the polyphenylene coating) is most likely attached to the LiFePO_4_ surface via O sites. Although the strength of the phenyl-O bond varies with the extent of surface lithiation, a strong chemical bond is expected under typical experimental and operational conditions as the surface is expected to be always deficient in Li to some extent. However, if the surface were to be completely lithiated, that is, by over-discharging a battery, the bonding of the coating to the surface could be disrupted because the bond strength would be weakened considerably and the preferred bonding site would shift from O to Fe.

## Discussion

It has long been desirable to obtain high-performance LiFePO_4_ particles with sizes approaching micrometres^24^,^25^,^26^,^27^. In this way, a high volumetric energy density of the Li-ion batteries resulted from a high tap density of micron-sized LiFePO_4_ particles can be achieved, which is critically important for electric vehicle applications^1^. Practically, it has been difficult to coat large LiFePO_4_ particles with uniform carbon coatings by calcination of carbon sources (usually organic small or macro molecules) and the preformed LiFePO_4_ particles, possibly because of the inhomogeneous mixing of carbon sources and the large LiFePO_4_ particles, which frequently results in phase-separated mixture of carbon and LiFePO_4_. On the other hand, simultaneous calcination of carbon sources and LiFePO_4_ precursors often produces only nano-sized carbon-coated LiFePO_4_ particles, because the formation of carbon phase inhibits the further growth of the LiFePO_4_ phase, which has been proved in numerous literatures on the synthesis of nano-LiFePO_4_ (refs 3, 4). Furthermore, as pointed out by Zhou and co-workers^16^, carbon coatings on LiFePO_4_ produced by heat treatment tend to be irregular and not well connected to the particles, particularly for large LiFePO_4_ particles, and hence do not fully deliver the expected performance for battery applications. Actually, the difficulty encountered when coating large LiFePO_4_ particles with a uniform carbon coating by calcination has motivated us to find alternatives to coat large LiFePO_4_ particles with other conducting materials in the first place, as exemplified in this work.

To demonstrate the importance of the conductive coatings (either carbon or polymer coatings) on large LiFePO_4_, we have tested the electrochemical performance (Supplementary Fig. 9) of bare LiFePO_4_ by preparing cathode of binder+LiFePO_4_ (1:9 wt/wt). To compare the performances between polyphenylene- and carbon-coated LiFePO_4_, we prepared and tested the cathode of binder+polyphenylene-coated LiFePO_4_ (1:9 wt/wt), and the cathode of binder+carbon additive+LiFePO_4_ (1:1:8 wt/wt) (Fig. 4; Supplementary Fig. 10). These results highlight the importance of the high-quality conducting coatings on the electrochemical performance of large pristine LiFePO_4_ particles, and that for improving the performance of micron-sized LiFePO_4_ particles, polyphenylene clearly outperforms the typical conducting carbon.

On the basis of the electrochemical measurement results of pristine, carbon-coated and polymer-coated LiFePO_4_ (Fig. 4; Supplementary Figs 9 and 10), and particularly on the fact that large LiFePO_4_ usually could not be easily coated with high-quality carbon coatings and therefore could only exhibit limited performance even in the presence of conducting carbon^24^,^25^,^26^,^27^, we attributed the improved electrochemical performance of polymer-coated large LiFePO_4_ particles to the intimate bonding between LiFePO_4_ and polyphenylene, a result of the surface-initialized electrografting of a diazonium salt, which is also supported by our DFT calculations. Another factor that might attribute to the improved performance of polymer-coated LiFePO_4_ is that Li^+^ diffusion coefficient in polyphenylene (∼1.32 × 10^−8^ cm^2^ s^−1^)^56^ is higher than in amorphous carbon coatings (∼9.0 × 10^−11^ m^2^ s^−1^)^57^. At higher rates, the Li^+^ diffusion within the coatings that separate the LiFePO_4_ particles and Li^+^ containing electrolytes become crucially important, because better Li^+^ ion conducting coatings can ensure a rapid exchange of Li^+^ ions between the LiFePO_4_ phase and the liquid Li^+^ electrolyte.

In summary, the energy capabilities of micron-sized LiFePO_4_ have been unlocked in this work. Using the intrinsic reducing power of LiFePO_4_ towards a diazonium salt of C_6_H_5_N_2_^+^BF_4_^−^, a covalently bonded conducting polymer coating of polyphenylene can spontaneously form on pristine LiFePO_4_. The reaction mechanism has been studied in a detailed way by a range of complementary techniques coupled with theoretical calculations. More importantly, we have shown that the standard carbon coating generated by pyrolysis reaction can be substituted by the polymer coatings without emission of volatile organic compounds. Moreover, the polymer-coated LiFePO_4_ can be used directly as ‘carbon additive free' electrodes with desired electrochemical performance for rechargeable Li-ion batteries. Combined, the method reported here represents a potential replacement of the industrial standard of carbon coating LiFePO_4_ with the spontaneous formation of conducting polymers from diazonium salt reactions.

## Methods

### Procedure

Carbon-free LiFePO_4_ powder was prepared according to a published procedure^18^. In brief, the stoichiometric amount of precursors of FePO_4_ (H_2_O)_2_ and Li_2_CO_3_ were thoroughly mixed together in isopropanol. After drying, the blend was heated at 700 °C under reducing atmosphere. The obtained LiFePO_4_ particle has an average size of 1.01 μm (Supplementary Table 3). C_6_H_5_N_2_^+^ BF_4_^−^ was synthesized as follows^31^. A total of 0.01 mol of newly distilled aniline was dissolved in 50 ml solution of 0.1 M of HCl. After cooling the solution at 0 °C with ice, a concentrated solution of NaNO_2_ (0.015 mol) in water was added for 20-min reaction, then 0.012 mol NaBF_4_ was added to precipitate the obtained diazonium cations. After filtration, the product was washed successively with cold water and ether. The powder was dried and kept in a freezer at −18 °C. The reactions of LiFePO_4_ and C_6_H_5_N_2_^+^BF_4_^−^ with different molar ratios were conducted in acetonitrile. After reaction, the obtained products were subjected to a centrifugation also in acetonitrile. The supernatant after centrifuge was collected and examined by ultraviolet–visible to ensure the complete removal of the possible non-surface confined polymer and the starting material. The electrochemical properties of polyphenylene-LiFePO_4_ were determined with CR2032-type coin cells using metallic lithium as the anode. The cathode was made by coating polyphenylene-LiFePO_4_ and a solution of PVDF (Kynar 2801; 90:10 wt/wt) in *N*-methylpyrrolidone onto Al foil.

### Characterization

Electroreduction of C_6_H_5_N_2_^+^ BF_4_^−^ was conducted in an air-tight, three-compartment glass cell with valves to control the gas inlet and outlet. A polycrystalline Au disk electrode (diameter 2.0 mm, CHI Inc.) was used as the working electrode and was polished with 0.05 μm alumina slurry before use. A partially delithiated Li_1−*x*_FePO_4_ (*x*=0.1) coated on stainless steel mesh (LiFePO_4_:Super P:PVDF 80:10:10 wt/wt) was used as the reference electrode. All electrochemical measurements were carried out using a Biologic VMP3 electrochemical workstation. The conductivity of the coated material was measured in a D41−11C/ZM four-probe resistivity tester. The chemical structure of C_6_H_5_N_2_^+^BF_4_^−^ was checked by ^1^H NMR (Avance III 400, Bruker) and ESI-MS(Quattro Premier XE system, Waters). PXRD was carried out using a STOE STADI/P diffractometer operating in transmission mode with a primary beam monochromator and position-sensitive detector. Fe Kα1 radiation (*λ*=1.936 Å) was employed. The details of online mass spectrometry were reported elsewhere^39^,^40^. The FTIR analysis was carried out with a Nicolet 6700 FTIR in transmission mode. Elemental analyses were performed using a Vario EL analyser. TEM images were recorded with JEOL JEM-2100F TEM operating at 200 kV. The experimental details for annular bright-field scanning TEM and high-angle annular scanning TEM for LiFePO_4_ were reported elsewhere^45^.

### Theoretical calculations

Periodic DFT calculations were performed using the Perdew-Burke-Ernzerhof functional and the projected augmented wave method as implemented in the Vienna Ab initio Simulation Package (version 5.3)^58^. The Kohn–Sham valence states (Fe(3*d*4*s*), Li(2*s*2*p*), O(2*s*2*p*), P(3*s*3*p*), C(2*s*2*p*) and H(1*s*)) were expanded in a plane wave basis up to a kinetic energy of 400 eV. As the electronic structures of both LiFePO_4_ and FePO_4_ are sensitive to electron correlation effects, the DFT+U approach was used^59^. The optimized U-values and lattice parameters for the bulk LiFePO_4_ and FePO_4_ were taken from Zhou *et al*.^60^. The electronic energies of LiFePO_4_ and FePO_4_ depend strongly on the magnetic state of the Fe atoms^61^. In accord with previous investigations, we found DFT+U to favour high-spin ferromagnetic and antiferromagnetic states over other spin orderings. As the binding energies of the phenyl radical on the LiFePO_4_(010) and FePO_4_(010) surfaces were observed to be reasonably insensitive to ferromagnetic/antiferromagnetic ordering of the Fe ions, we report here results for high-spin ferromagnetic states, where the site projected atomic magnetic moments of Fe remain essentially identical to their computed bulk values (4.3 and 3.7 μ_B_ for FePO_4_ and LiFePO_4_, respectively). Following relaxation of the bulk atomic positions, symmetric (010) slabs were constructed according to the procedure outlined in ref. 51. The dimensions of each slab correspond to 1**a** × 2**b** × 2**c** of the bulk lattice vectors (where **a**=10.42, **b**=6.07 and **c**=4.75 Å for LiFePO_4_ and **a**=9.99, **b**=5.88 and **c**=4.87 Å for FePO_4_), yielding 16 formula units per unit cell. Vacuum space (13 Å) was added along the **b** direction. The surface Brillouin zone was sampled with a 3 × 1 × 3 k-point mesh, which was confirmed to converge the total energies of both surfaces to within 3 meV per formula unit. For FePO_4_(010), the atomic positions of the top layer of Fe and P atoms along with all the O atoms coordinated to them (including subsurface O atoms) were relaxed, while holding the coordinates of the remaining atoms frozen in their bulk positions. For LiFePO_4_(010), the same layer of Fe, P and O atoms as in FePO_4_(010) was relaxed, as well as the top two layers of Li atoms. The vacancy model was created by removing one of the two top-layer Li atoms in the surface unit cell that we used for LiFePO_4_(010), with the surface relaxed again. The phenyl adsorbate was fully relaxed. All geometry relaxations were considered converged once the force in each relaxed degree of freedom fell below 0.03 eV Å^−1^. The adsorption energy of phenyl was calculated as Δ*E*_ads_=*E*_total_−*E*_surface_−*E*_phenyl_. Including the semi-core states of Fe and Li in the valence (Fe(3s3p3d4s), Li(1s2s2p)) and increasing the kinetic cutoff energy to 500 eV were verified to changed adsorption energies by <0.1 eV on all three surfaces.

## Additional information

**How to cite this article:** Guo, L. *et al*. Unlocking the energy capabilities of micron-sized LiFePO_4_. *Nat. Commun*. 6:7898 doi: 10.1038/ncomms8898 (2015).

## Supplementary Material

Supplementary Information Supplementary Figures 1-12 and Supplementary Tables 1-3

## Figures and Tables

**Figure 1:**
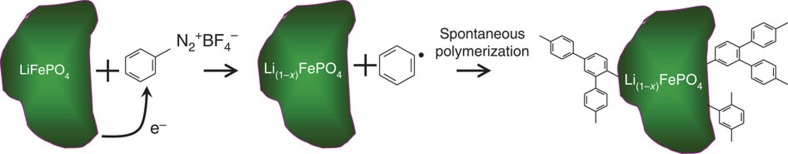
Schematic illustration of the reaction of LiFePO_4_ and C_6_H_5_N_2_^+^BF_4_^−^. The diazonium cations are reduced to phenyl radicals by electrons from LiFePO_4_ particles, while at the same time LiFePO_4_ is oxidized to its partially delithiated state of Li_1−x_FePO_4_. The reactive phenyl radicals bond to the surface of Li_1-x_FePO_4_ forming conducting polyphenylene coatings.
