# Supplementary material for: Unlocking the energy capabilities of micron-sized LiFePO4
Source: Nat Commun. 2015 Aug 3;6:7898. doi: 10.1038/ncomms8898 (PMC4532849; doi:10.1038/ncomms8898)
Supplement: Supplementary Information — Supplementary Figures 1-12 and Supplementary Tables 1-3 [file ncomms8898-s1.pdf]

Supplementary Figures

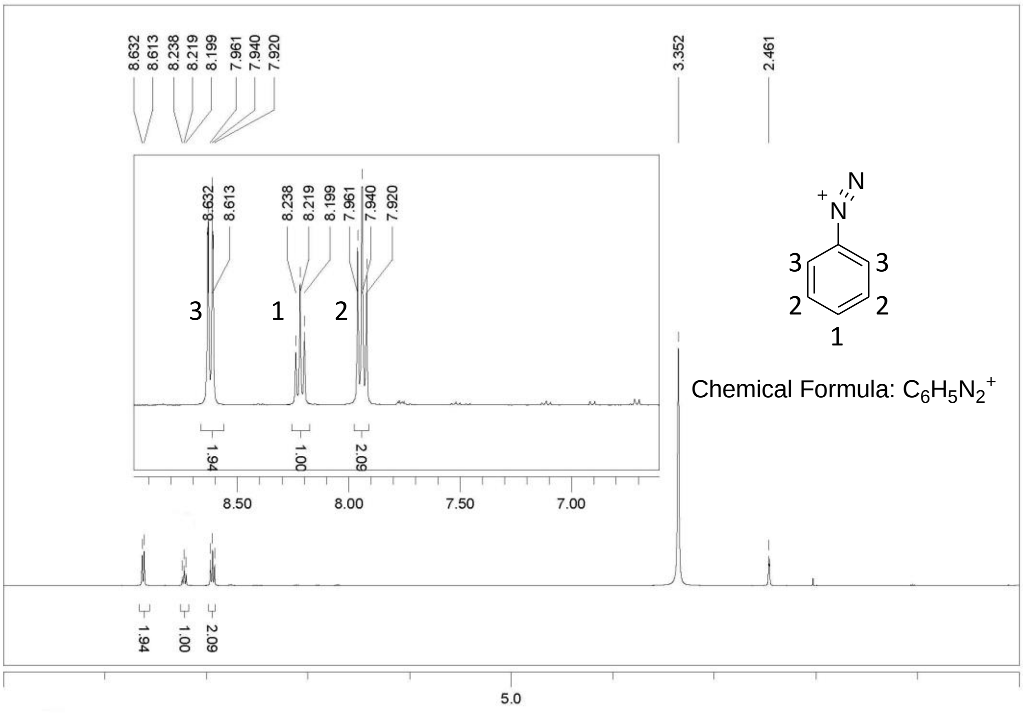

Supplementary Fig. 1 ^{1}H-NMR of $C_6H_5N_2^+BF_4^-$ in DMSO-d6.

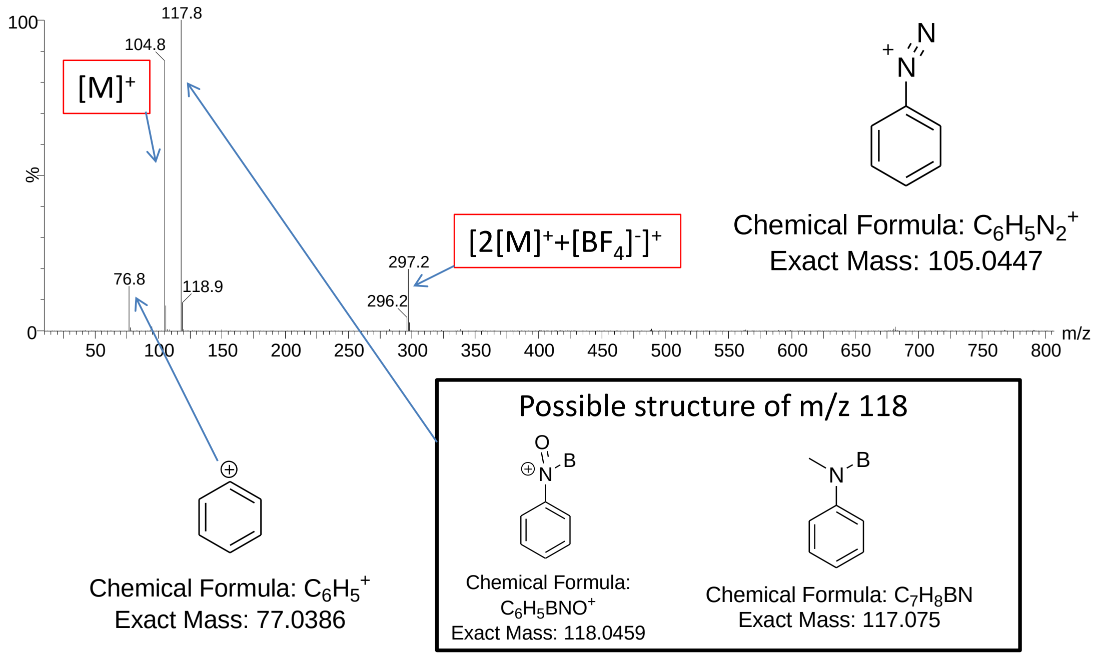

Supplementary Fig. 2 ESI-MS of $C_6H_5N_2^+BF_4^-$ dissolved in methanol.

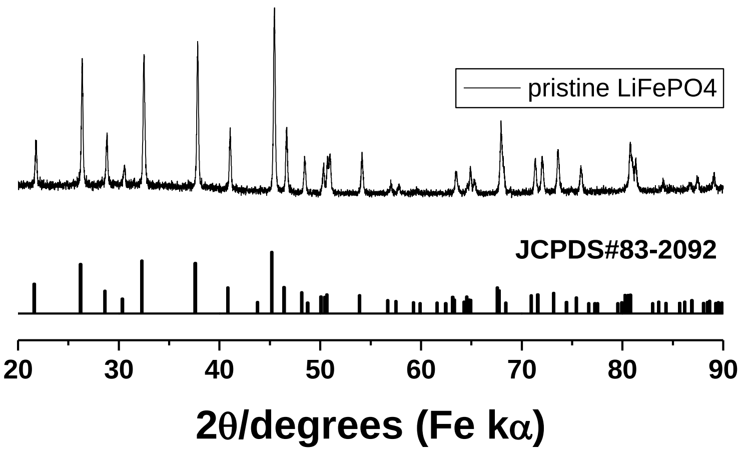

Supplementary Fig. 3 PXRD of as-synthesized carbon free $LiFePO_4$.

(a)

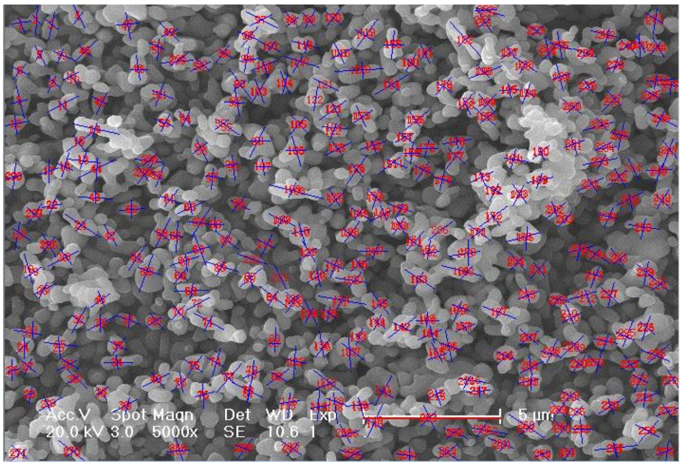
Acc.V Spot Magn Det WD Exp
20.0 kV 3.0 5000x SE 10.6 1
5 µm

(b)

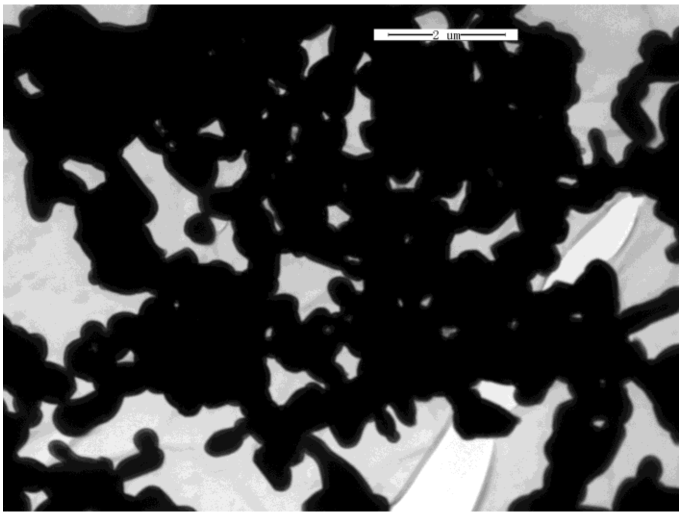
2 um

(c)

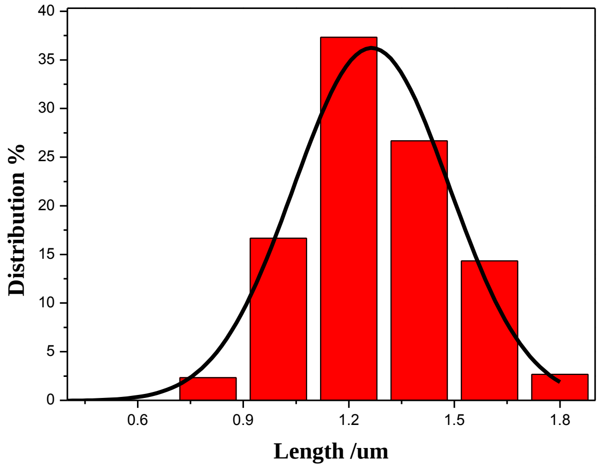
Distribution %
Length /um
0
5
10
15
20
25
30
35
40
0.6
0.9
1.2
1.5
1.8

(d)

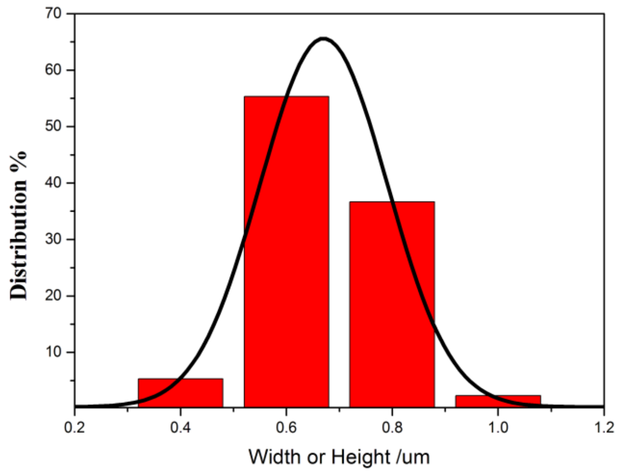

(e)

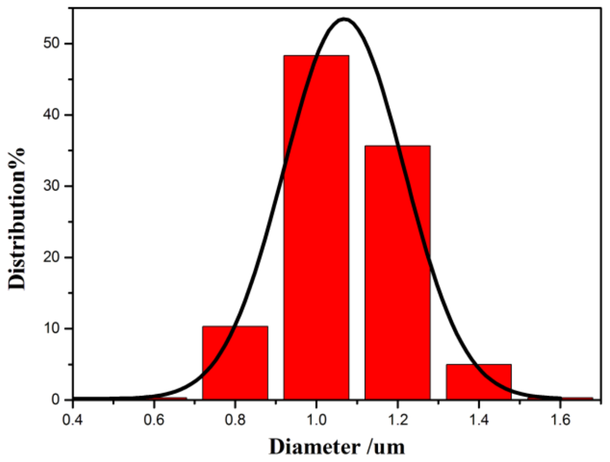

Supplementary Fig. 4 (a) SEM, (b) TEM micrographs, (c) the length and (d) the width/ height distribution of the cuboidal $LiFePO_4$, and (e) the diameter distribution of the spherical $LiFePO_4$. The statistic results were based on the analysis of SEM image in (a).

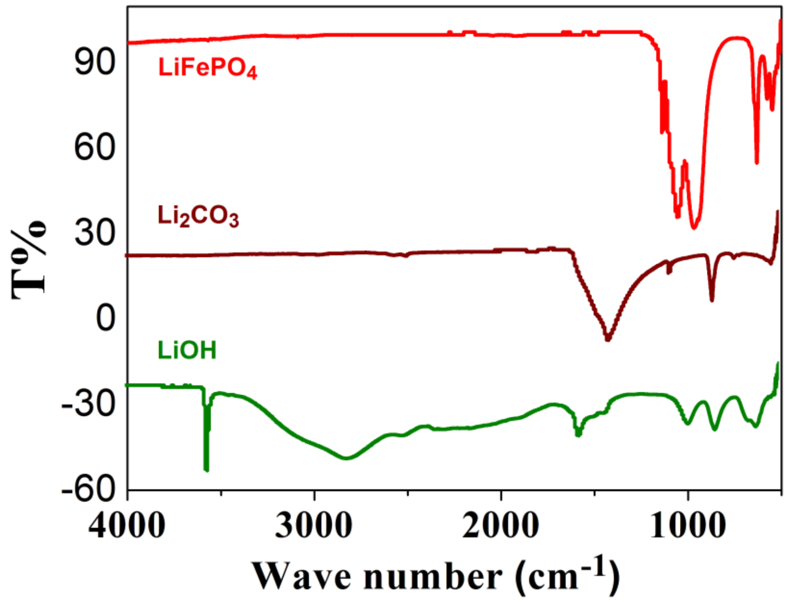

Supplementary Fig. 5 FTIR spectrum of pristine $LiFePO_4$. For comparison, FTIR spectra of Li_2CO_3 and LiOH have also been collected and presented.

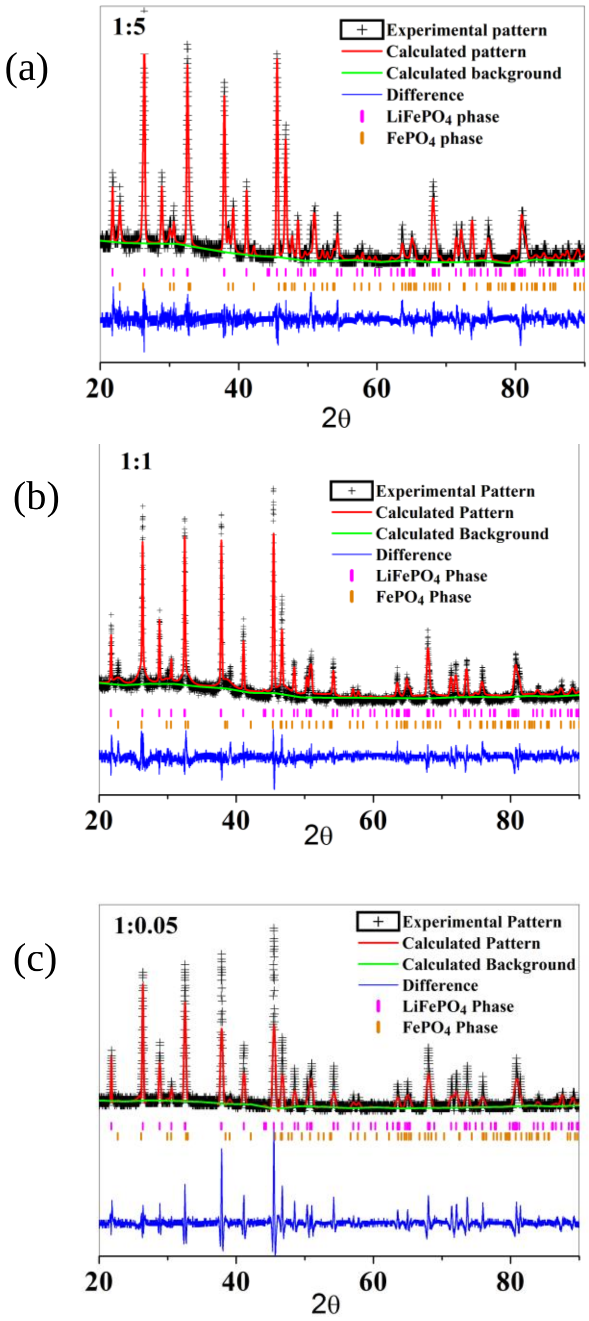

Supplementary Fig. 6 Rietveld refinement results of the PXRD data of $LiFePO_4$ samples after reaction with $C_6H_5N_2^+BF_4^-$ with different molar ratio of (a) 1:5, (b) 1:1 and (c) 1:0.05.

(a) (b) (c) (d) (e)

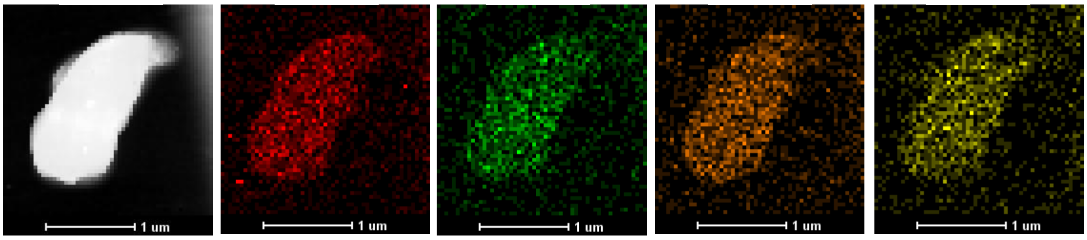

Supplementary Fig. 7 (a) High-angle annular dark-field scanning transmission (HAADF-STEM) image of a single $LiFePO_4$ particle and its elementary mapping by EDX including (b) C $_{ka}$, (c) Fe $_{ka}$, (d) O $_{ka}$ and (e) P $_{ka}$. The sample was obtained by the reaction of $LiFePO_4$ and $C_6H_5N_2^+BF_4^-$ with a molar ratio of 1:0.05. The obviously enhanced signal of carbon observed in image (b) suggests the presence of polymer formed on $LiFePO_4$. However, EDX does not have the ability to distinguish $LiFePO_4$ and $FePO_4$ phases within a single particle due to its limited energy resolution. The co-existence of $LiFePO_4$ and $FePO_4$ phases has been proved by the ABF-STEM in **Supplementary Fig. 8**.

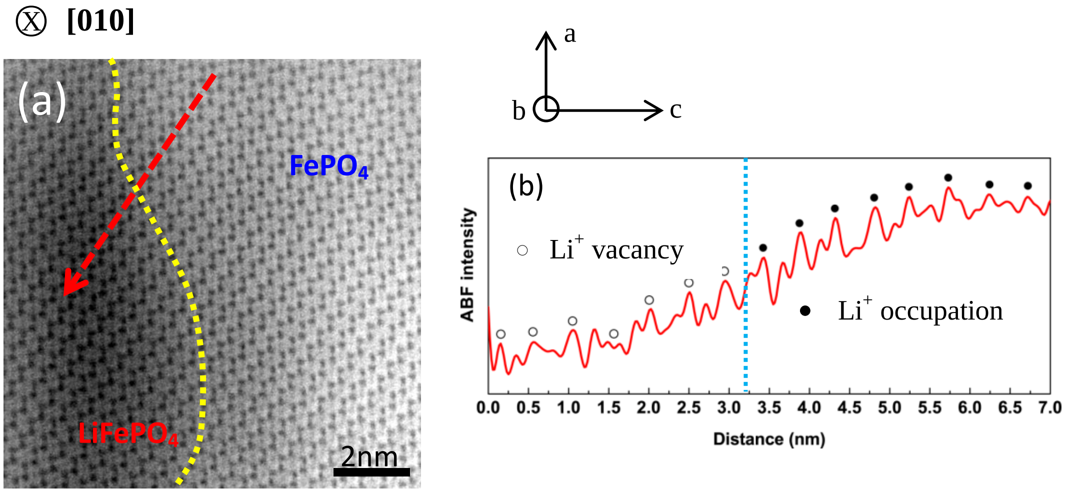

Supplementary Fig. 8 (a) Annular-bright-field scanning transmission electron microscopic (ABF-STEM) image of the interface between $LiFePO_4$ and $FePO_4$ phases. The interface is indicated by a dotted yellow line. (b) A line profile corresponding to the dotted red arrow in (a) shows the ABF intensity across the interface, with zero distance being the start of the red arrow. In the ABF line profile, image contrast of the dark dots is inverted and displayed as peaks. The sample was obtained by the reaction of $LiFePO_4$ and $C_6H_5N_2^+BF_4^-$ with molar ratio of 1:0.05.

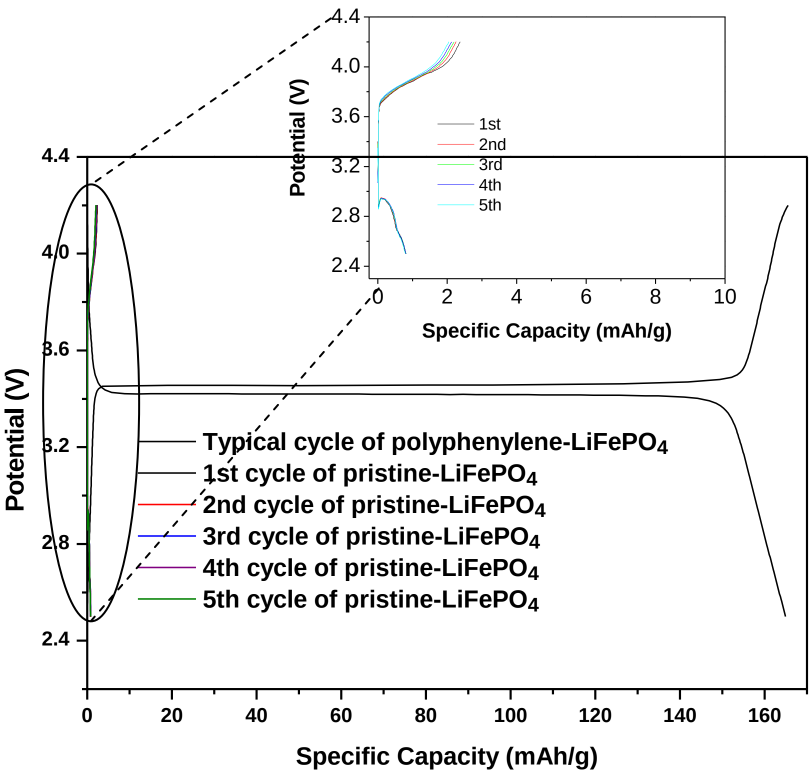

Supplementary Fig. 9 Charge/discharge curves of a cathode containing $LiFePO_4$ and PVDF (90:10 wt/wt), in which the $LiFePO_4$ was treated in the absence of $C_6H_5N_2^+BF_4^-$. For comparison, typical charge/discharge curve of the polyphenylene-$LiFePO_4$ has also been presented. Inset shows the zoom-in of the electrochemical performance of the uncoated $LiFePO_4$.

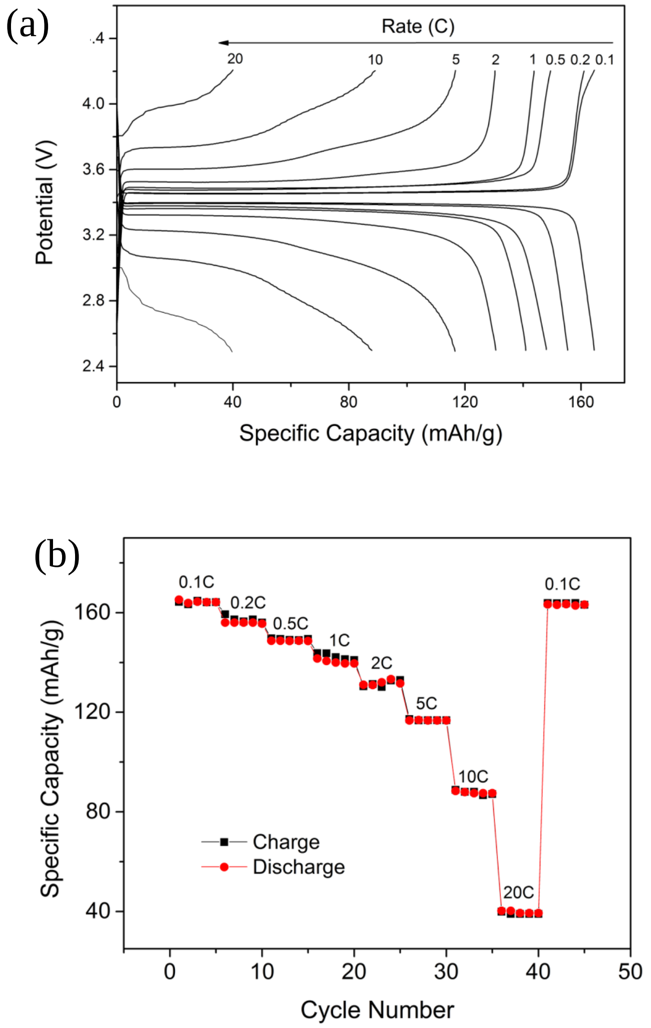

Supplementary Fig. 10 (a) Charge/discharge curves of cathode made of $LiFePO_4$, Super P and PVDF (80:10:10 wt/wt) at various rates from 0.1 to 20 C; (b) charge/discharge capacity vs. cycle number.

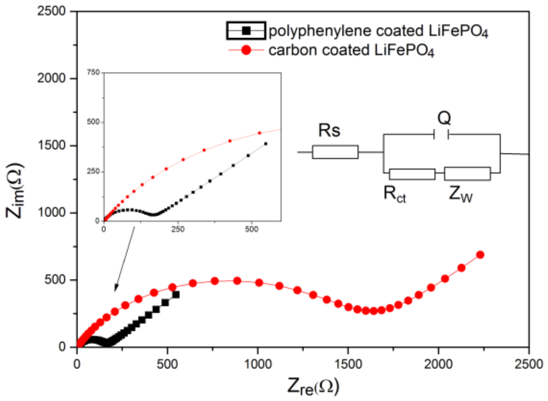

Supplementary Fig. 11 EIS spectra of polyphenylene and carbon-coated $LiFePO_4$ with the equivalent circuit as an inset. EIS spectrum of pristine $LiFePO_4$ has not been successfully acquired due to the poor conductivity of the composite cathode of pristine $LiFePO_4$ and PVDF binder (90:10 wt/wt). As an alternative, composite cathode of $LiFePO_4$, Super P and PVDF binder (80: 10: 10 wt/wt) was prepared and examined.

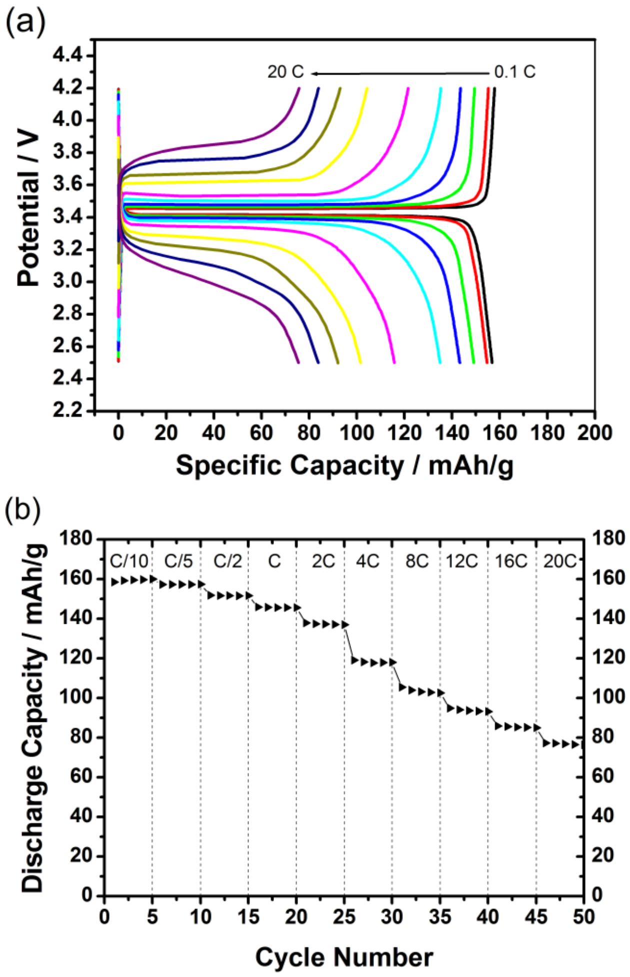

Supplementary Fig. 12 (a) Charge/discharge curves of polyphenylene-$LiFePO_4$/PVDF (90:10 wt/wt) at various rates from 0.1 to 20 C; (b) discharge capacity vs. cycle number. The electrochemical tests were conducted at 0 ^{o}C.

Supplementary Tables

Supplementary Table 1 Tabulated Rietveld refinement results of the PXRD data of the reaction of $LiFePO_4$ and $C_6H_5N_2^+BF_4^-$ with different reaction ratios. As shown in the table, Rwp and Rp factors of the 1:0.05 reaction ratio are large and are hard to be precisely fitted. Therefore, the elementary analysis and online mass spectrometric results, instead of the refinement one, were discussed in the main text.

Samples	1:0.05	1:1	1:5
$LiFePO_4$			
Ratio (%)	89.81	56.07	51.59
a (Å)	10.3192	10.3238	10.3186
b (Å)	6.0055	6.0052	5.9988
c (Å)	4.6940	4.6942	4.6939
V (Å^3)	290.892	291.024	290.551
$FePO_4$			
Ratio (%)	10.19	43.93	48.41
a (Å)	9.8189	9.8282	9.8213
b (Å)	5.8037	5.8230	5.7934
c (Å)	4.7318	4.7591	4.7655
V (Å^3)	269.649	273.721	271.151
Rwp (%)	9.26	4.62	2.74
Rp (%)	6.01	3.43	2.06

Supplementary Table 2 Chemical diffusion coefficients of Li in $Li_{1-x}FePO_4$ obtained from EIS at various Li contents. Technical details can be found in the literature: Tang, K., Yu, X., Sun, J., Li, H. & Huang, X. Kinetic analysis on $LiFePO_4$ thin films by CV, GITT and EIS. *Electrochim. Acta.* **56**, 4869-4875 (2011).

X in $Li_{1-X}FePO_4$	polyphenylene-$LiFePO_4$ ($cm^2\ s^{-1}$)	C-$LiFePO_4$ ($cm^2\ s^{-1}$)
0.1	7.86×10^{-14}	6.65×10^{-14}
0.2	6.23×10^{-14}	5.01×10^{-14}
0.3	3.54×10^{-14}	3.38×10^{-14}
0.4	8.23×10^{-15}	5.81×10^{-15}
0.5	1.76×10^{-16}	1.01×10^{-16}
0.6	5.30×10^{-15}	4.73×10^{-15}
0.7	6.39×10^{-14}	5.24×10^{-14}
0.8	3.72×10^{-14}	2.13×10^{-14}
0.9	4.24×10^{-14}	3.65×10^{-14}
1	6.44×10^{-14}	5.09×10^{-14}

Supplementary Table 3 The statistical report of the particle size based on supplementary Fig. 4

Counts				Percentage %		
**Distr./μm**	**Length**	**Width or Thickness**	**Diameter**	**Length**	**Width or Thickness**	**Diameter**
0.4-0.6	0	16	0	0	5.33	0
0.6-0.8	0	166	1	0	55.33	0.33
0.8-1.0	7	110	31	2.33	36.67	10.33
1.0-1.2	50	7	145	16.67	2.33	48.34
1.2-1.4	112	1	107	37.33	0.34	35.67
1.4-1.6	80	0	15	26.67	0	5
1.6-1.8	43	0	1	14.33	0	0.33
1.8-2.0	8	0	0	2.67	0	0
2.0-2.2	0	0	0	0	0	0
Sum.	300	300	300	100	100	100
Mean/μm	1.20	0.67	1.01			
